# Case report: Translational treatment of unresectable intrahepatic cholangiocarcinoma: Tislelizumab, Lenvatinib, and GEMOX in one case

**DOI:** 10.3389/fonc.2024.1428370

**Published:** 2024-07-15

**Authors:** He-wei Zhang, Hai-bo Yu

**Affiliations:** Department of Hepatobiliary and Pancreatic Surgery, The Dingli Clinical Institute of Wenzhou Medical University (Wenzhou Central Hospital), Wenzhou, Zhejiang, China

**Keywords:** advanced intrahepatic cholangiocarcinoma, chemotherapy, immunotherapy, targeted therapy, case report

## Abstract

**Background:**

Intrahepatic cholangiocellular carcinoma (ICC) is one of the most common invasive malignancies. Currently, ICC is treated with radical surgical resection. However, the majority of patients are diagnosed at an advanced stage, making surgery ineligible for them.

**Case presentation:**

We present a case of advanced ICC, which could not undergo radical surgery due to tumor invasion of liver blood vessels. The gemcitabine and oxaliplatin (GEMOX) regimen combined with Tislelizumab immunotherapy and Lenvatinib targeted therapy for 8 cycles resulted in significant tumor shrinkage significantly and the vascular invasion disappeared. CA19–9 levels were reduced to normal levels. Partial remission and successful tumor transformation were achieved. The patient underwent a successful radical surgical resection, including cholecystectomy, resection of liver segments IV, V, and VIII, as well as a regional lymphatic dissection procedure, resulting in complete pathological remission.

**Conclusion:**

Tumor-free surgical margins (R0) resection of patients with advanced ICC after combination of immune, targeted and chemotherapy is rare, and there are almost no cases of complete postoperative remission. The GEMOX regimen in combination with Tislelizumab and Lenvatinib has a good antitumor efficacy and safety profile, and may be a feasible and safe translational treatment option for advanced ICC.

## Introduction

Cholangiocarcinoma (CCA) is an aggressive cancer of the biliary system and the second most common primary liver tumor. Its poor prognosis stems from its propensity for extensive metastasis, heightened resistance to pharmacological interventions, and the absence of efficacious therapeutic alternatives (1). Over the past decade, the occurrence rate of CCA surged from 0.67 cases per 100,000 individuals in 2007 to 1.40 cases per 100,000 individuals in 2016 (2). Intrahepatic cholangiocarcinoma (ICC), a subtype of CCA, originates from the epithelial cells of the intrahepatic bile ducts and has a 5-year overall survival rate of approximately 18% (3). The only treatment for ICC is surgical resection in order to achieve R0. However, due to its insidious onset and lack of typical clinical symptoms and biomarkers, it is often diagnosed only at advanced local or distant metastases. From 1983 to 2010, data from the SEER (Surveillance Epidemiology and End Results) database indicate that merely 15% of patients diagnosed with confirmed ICC were eligible for radical surgical resection (4). Data registered by the European Network for CCA Research (ENSCCA) between 2010 and 2019 validate these results (5). The emergence of enhanced chemotherapeutic, targeted, and immunological agents has ushered in a new era, where a multidisciplinary strategy comprising surgical interventions, systemic or localized therapies, and radiotherapy presents viable treatment avenues for individuals grappling with advanced ICC.

It wasn’t until 2010 that gemcitabine/cisplatin (Gem/CDDP) chemotherapy was demonstrated to be an efficacious first-line regimen. To date, Gem/cisplatin (Gem/CDDP) chemotherapy is endorsed by the National Comprehensive Cancer Network (NCCN) as the primary treatment option for advanced ICC. However, the overall effect of chemotherapy is limited, with a low objective effectiveness rate (ORR) and susceptibility to drug resistance (6, 7). Programmed cell death protein 1 (PD-1) and its ligand, programmed cell death 1 ligand 1 (PD-L1), serve as immune checkpoint proteins, exerting a suppressive effect on immune responses. In the treatment landscape, inhibitors targeting PD-1/PD-L1 have exhibited encouraging outcomes across diverse cancer types (8). As of now, PD-1 inhibitors have not received approval for first-line systemic therapy in biliary tract cancers, except for Pembrolizumab, which is indicated for mismatch repair-deficient (dMMR) or high microsatellite instability (MSI) tumors. Numerous investigations have assessed the therapeutic effectiveness of PD-1 inhibitors when combined with chemotherapy for advanced biliary tract cancer (9–11).The combination of PD-1 inhibitors with chemotherapy significantly improved the median OS in patients with advanced ICC compared to PD-1 inhibitors alone and chemotherapy alone (10). Lenvatinib, characterized as a multi-targeted tyrosine kinase inhibitor (TKI), has gained approval for managing unresectable hepatocellular carcinoma (HCC). The combination of Lenvatinib and Immune checkpoint inhibitors (ICIs) has yielded positive results and offers a broader perspective for HCC. In terms of ICC, the phase II clinical trial (NCT02579616) used Lenvatinib as a single agent in the treatment of unresectable biliary tract cancer, and the results showed promising antitumor activity (12). Based on the drug safety of the combination of Lenvatinib and ICIs, several recent studies have used the combination of Lenvatinib and ICIs in the treatment of advanced ICC. Xie et al. conducted an evaluation on the effectiveness of combining Lenvatinib with a PD-1 inhibitor in a cohort of 40 patients diagnosed with intrahepatic cholangiocarcinoma (ICC), who had experienced treatment failure with chemotherapy for advanced disease. Their findings demonstrated that the combination of Lenvatinib and PD-1 inhibitors exhibited notable efficacy in addressing advanced ICC following chemotherapy resistance (13).

Many studies have demonstrated the ability of chemotherapy combined with targeted and immunotherapy to alleviate advanced biliary tract cancer (BTC). However, there are few cases of complete remission after resection of advanced ICC conversion therapy (14). In this report, we present the case of a patient diagnosed with unresectable advanced intrahepatic cholangiocarcinoma (ICC) who achieved complete postoperative remission without recurrence following successful surgical R0 resection. The patient underwent the GEMOX regimen in combination with PD-1 inhibitors Tislelizumab and Lenvatinib as first-line treatment. This case offers valuable insights and guidance for the clinical management of advanced ICC.

## Case presentation

**Figure 1:**
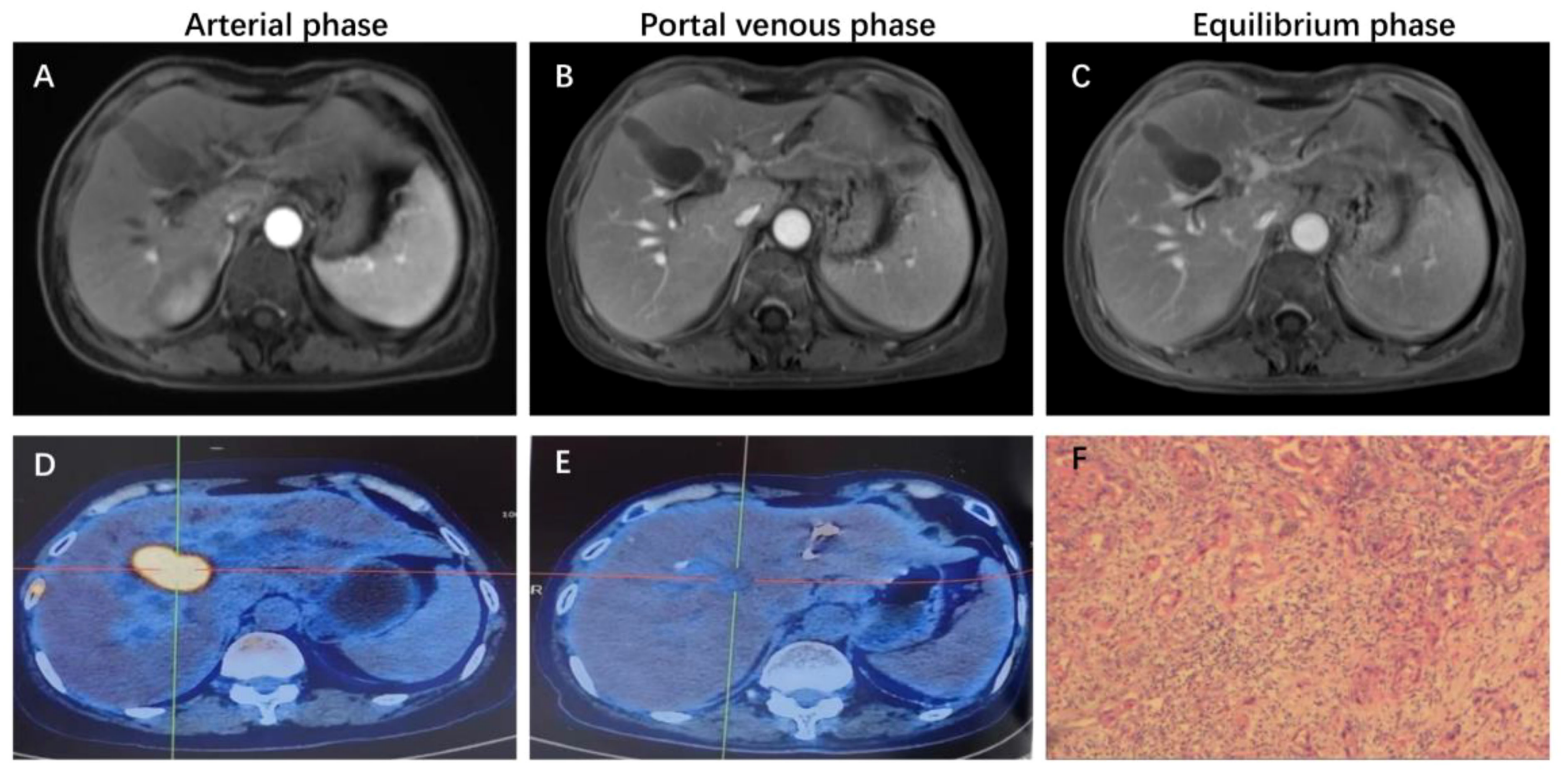
Representative MRI imaging and PET-CT maps at the end of 8 cycles of combination therapy. **(A-C)** At the end of the combined treatment, the liver enhanced MRI axial T1 weighted images of artery, portal vein and balance phase showed no obvious enhancement of the tumor. **(D)** Increased 18F-FDG metabolism of the mass is seen on PET-CT of the liver tumor before combination therapy. **(E)** significant decrease in 18F-FDG metabolism in the visible mass of liver tumor PET-CT at the end of combination therapy. **(F)** H&E staining results of liver tumor biopsy guided by ultrasound.

On 3 September 2022, a 51-year-old Chinese woman presented to our hospital with “20 days of jaundice in the whites of the skin and eyes”. On the 10th day of her jaundice, she underwent percutaneous transhepatic cholangial drainage and ultrasound-guided biopsy of the liver tumor on 23 August 2022 at an outside hospital. Pathological examination (**Figure 1F**) showed intrahepatic cholangiocarcinoma. Her occupation is worker, and her education level is primary school. There was a past history of breast cancer 12 years ago, which was cured. The patient’s medical history revealed no past or present alcohol consumption or hepatitis B infection. There were no tumors in her family. Physical examination of her revealed generalized jaundice of the skin, mucous membranes and sclera. She exhibited epigastric tenderness without rebound tenderness, lacked any significant abdominal mass, and showed no notable enlargement of the subclavian lymph nodes. Laboratory investigations revealed significant elevation of glycan chain antigen 199 (CA199) (589.7 KU/L, normal range 0–25 KU/L), glycan chain antigen 125 (CA125 (72.3 KU/L, normal range 0–23 KU/L), carcinoembryonic antigen (CEA) (25.3 ug/L, normal range 0–5 ug/L), alanine aminotransferase (91 U/L, normal range 7–40 U/L), aspartate aminotransferase (52 U/L, normal range 13–35 U/L), total bilirubin (93.5 µmol/L, normal range 0–21 µmol/L), elevated direct bilirubin (51.8 μmol/L, normal range 0–4 μmol/L, elevated indirect bilirubin level (41.7 μmol/L, normal range 0–21 μmol/L). Alpha-fetoprotein (AFP) and glycoconjugate antigen 125 (CA125) levels were in the normal range. Abdominal plain + enhanced CT showed hepatoportal occupancy, consideration of intrahepatic bile duct dilatation secondary to cholangiocellular malignancy. Abdominal MRCP showed multiple abnormal signals in the liver, marked dilatation of the intrahepatic bile ducts, and partial truncation of the bile ducts in the portal region. CTA of the hepatic artery, CTV of the portal vein, CTV of the hepatic vein, and CTV of the inferior vena cava showed intrahepatic cholangiocarcinoma with possible hepatoportal metastasis, with the lesion locally adhering to the right branch of the portal vein and the right hepatic artery, and the intrahepatic bile ducts were dilated. Based on these findings, we diagnosed ICC with intrahepatic vascular invasion. As per the 8th edition of the American Joint Committee on Cancer (AJCC) Cancer Staging Manual, the clinical TNM stage was identified as T2N0M0 (stage II).

## Treatment

**Figure 2:**
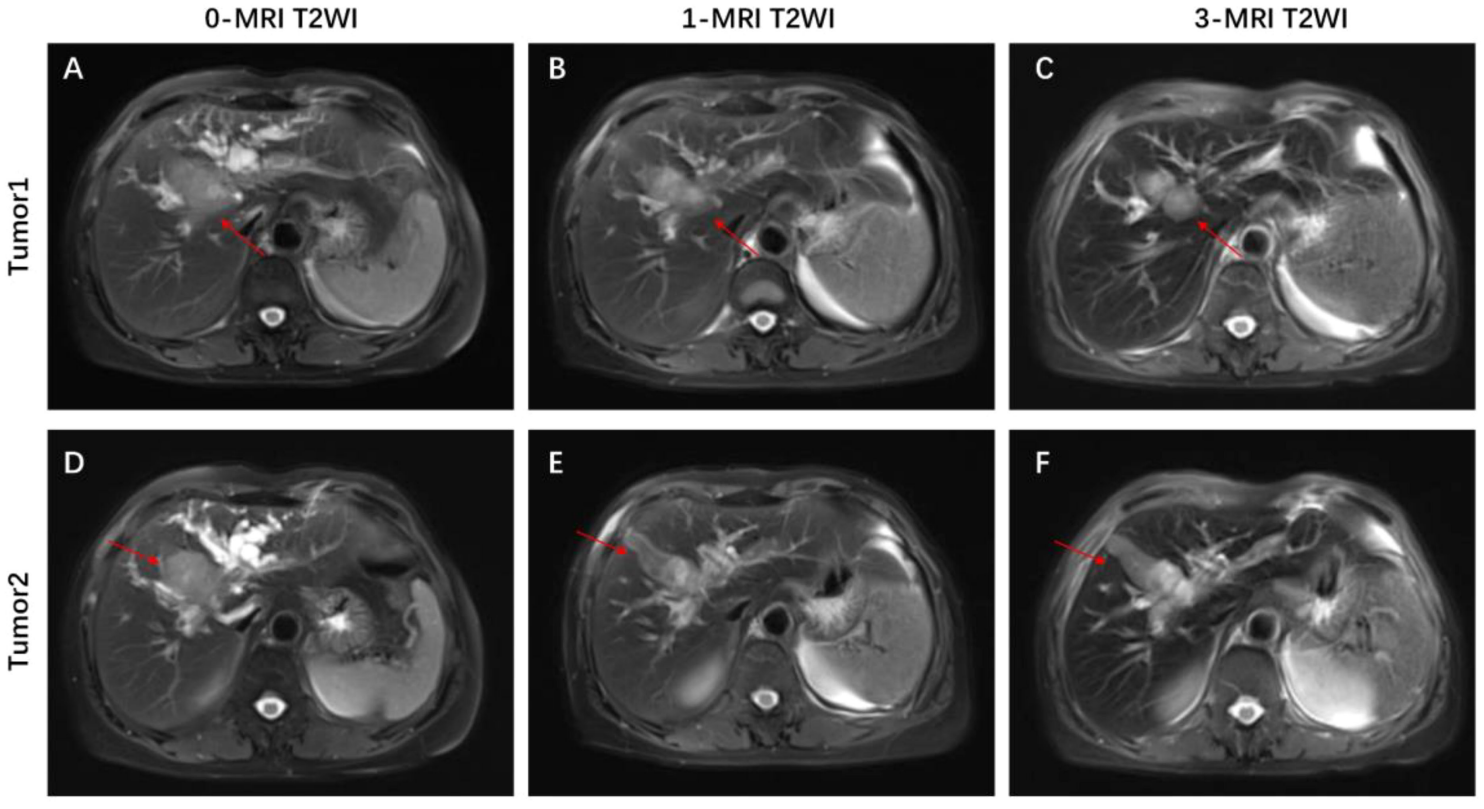
Representative MRI images showing changes in the tumor before and after combination therapy. **(A, D)** axial T2-weighted MRI images of the liver before combination therapy, the tumor consists of two parts. **(B, E)** Axial T2-weighted MRI image of the liver at 1 cycle of combination therapy, with a shrinkage of the mass near the hilar region and a change in the morphology of the other mass. **(C, F)** Axial T2-weighted MIR image of the liver at 3 cycles of combined treatment, with significant shrinkage of the mass near the hilar region.

**Figure 3:**
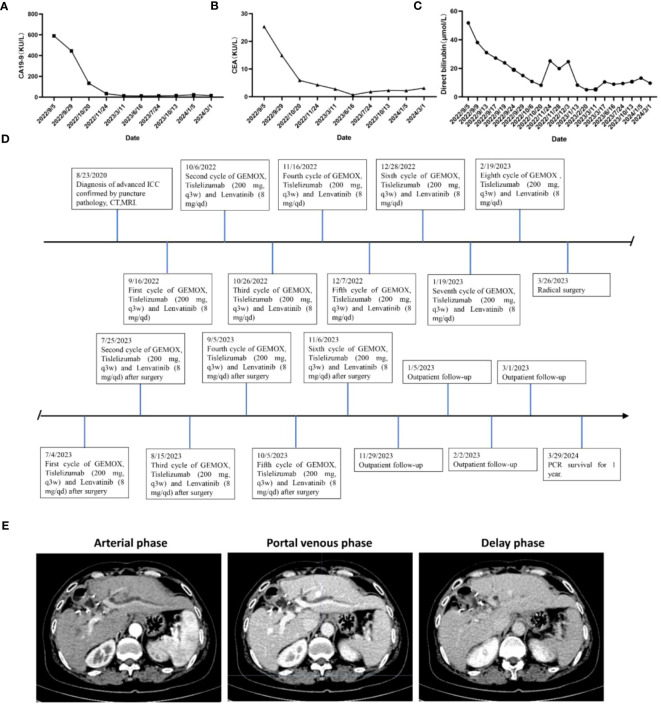
Changes in **(A)** CA19–9, **(B)** CEA expression levels and levels of **(C)** total bilirubin during combination therapy for advanced ICC. **(D)** Timeline of initial diagnosis, utilization of combination therapy, radical surgery, adjuvant therapy and subsequent follow-up. **(E)** Enhanced CT scan of liver 1 year after operation.

This patient was unable to undergo radical resection due to tumor invasion of intrahepatic vessels. After a multidisciplinary consultation, on 15 September 2022, the patient was initiated to receive GEMOX chemotherapy (gemcitabine 1000 mg/m2, iv, d1, d8/3w combined with oxaliplatin 125 mg/m2, iv, d2/3w) and anti-PD-1 immunotherapy (Tislelizumab 200 mg, iv, q3w) combined with targeted therapy (Lenvatinib 8 mg, po, qd) as a treatment regimen. Adverse events that occurred during treatment included decreased appetite, nausea, peripheral sensory neuropathy, thrombocytopenia, neutropenia and drug-induced liver injury. Recombinant human granulocyte stimulating factor was used to correct neutropenia in patients. Severe thrombocytopenia was ameliorated by platelet transfusion and recombinant human interleukin-11. We administered N-acetylcysteine (NAC) and Polyene Phosphatidylcholine injections to patients to improve drug-induced liver damage. In response to the nausea and vomiting that occurred during the treatment process, we provided patients with Palonosetron and Dexamethasone injections to improve symptoms. All adverse events related to immunological and chemotherapy treatments were effectively managed and resolved following the completion of treatment. After 1 cycle of conversion therapy, two nodules were found in the hilar region, the one near the hilar area was smaller than before (**Figures 2A, B**), and the other nodule was significantly altered in morphology and had a “pipe-like” shape (**Figures 2D, E**). After 3 cycles of conversion therapy, one nodule near the porta hepatis continued to shrink compared with the previous one (**Figure 2C**), and the other nodule showed no significant change (**Figure 2F**). After 8 cycles of treatment, abdominal CT showed that one nodule near the porta hepatis continued to shrink compared with the previous one, the other nodule showed no significant change, and the tumor showed no significant enhancement on enhanced magnetic resonance (**Figures 1A–C**), no tumor invasion of blood vessels was found. At the end of 8 cycles of combination therapy, the metabolism of 18F-FDG in the liver tumor decreased significantly by PET-CT (**Figures 1D, E**), suggesting that the treatment was effective. CA19–9 levels (**Figure 3A**) and CEA levels (**Figure 3B**) showed a continuous decreasing trend during the treatment. Tumor efficacy was evaluated according to the mRECIST criteria and was judged as partial remission (PR). The changes of total bilirubin during the whole treatment process are shown in **Figure 3C**. The patient concluded the final cycle of conversion therapy on February 19, 2023. One month after discontinuation of combination therapy, the patient’s routine hematology showed mild anemia, with no significant abnormalities in coagulation or renal function test results.CA199, CA125, CEA, AFP and CA125 were in the normal range. Alanine aminotransferase (79 U/L, normal range 7–40 U/L), aspartate aminotransferase (60 U/L, normal range 13–35 U/L), total bilirubin (13.9 µmol/L, normal range 0–21 µmol/L), elevated direct bilirubin (5.3 μmol/L, normal range 0–4 μmol/L, elevated indirect bilirubin level (8.6 μmol/L, normal range 0–21 μmol/L). On 26 March 2023, the patient underwent hepatic resection of liver segments IV, V and VIII + cholecystectomy + regional lymph node dissection.

## Operative findings

**Figure 4:**
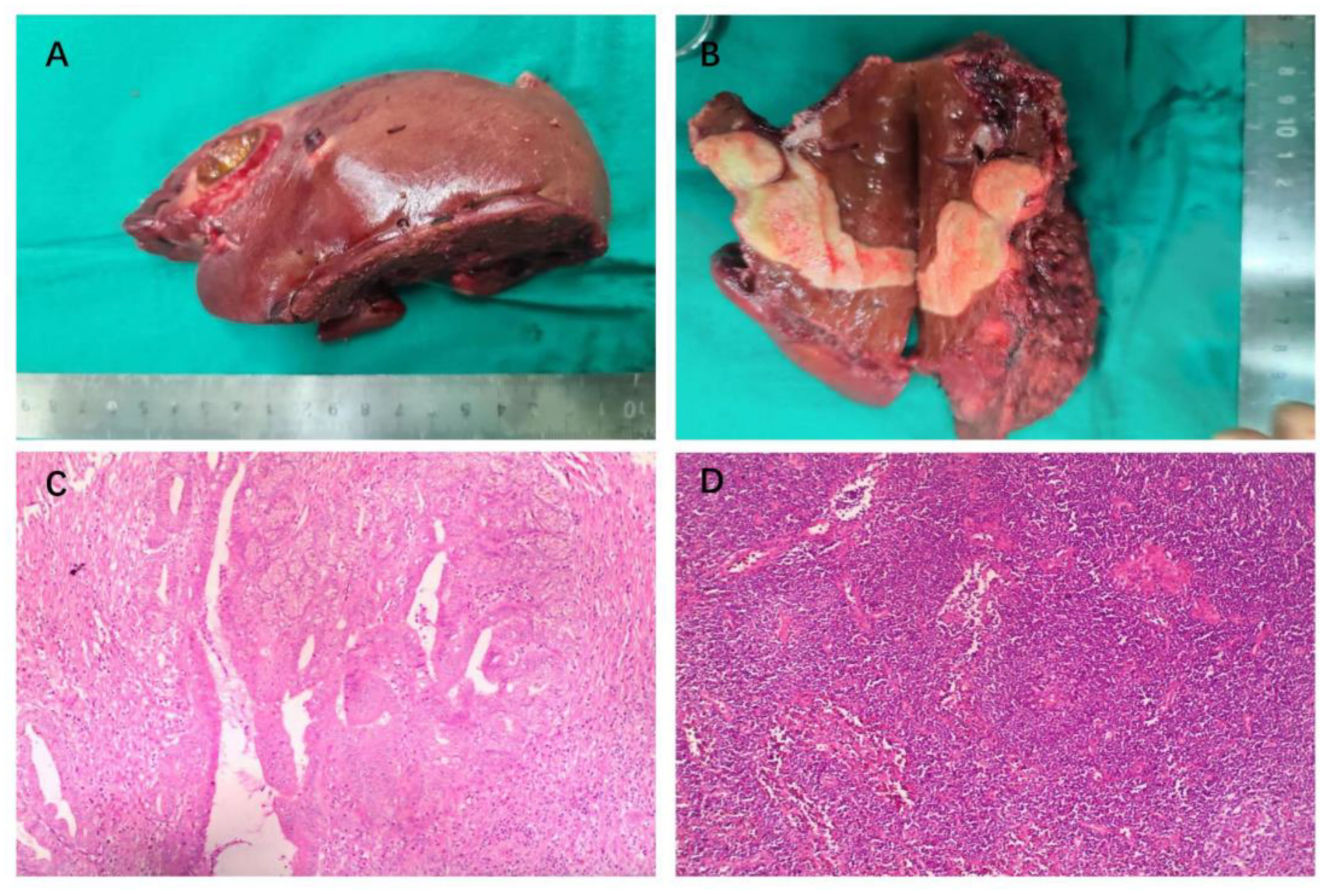
Intraoperative gross pathology specimens and HE staining of radical resection in a patient with advanced ICC. **(A)** Resection specimens of liver segments IV, V and VIII, measuring about 14x7x6 cm. **(B)** The visible tumor consists of two parts, measuring about 6.5 x 2.5 x 2.5 cm and 2.6 x 2 x 0.8 cm. **(C)** H&E staining shows extensive necrosis (>95%) of the tumor tissue, with predominantly fibrotic and inflammatory cellular infiltration, and no tumor-active components were seen. **(D)** H&E staining showed chronic inflammation of gallbladder.

A reverse L-shaped incision was made in the right upper abdominal region to perform the dissection. The surface of the liver was smooth, and ultrasound examination revealed two nodules in the porta hepatis with diameters of about 3.0 and 1.8 cm. Enlarged lymph nodes with hard texture could be found in the hepatoduodenal ligament, posterior duodenum, adjacent to the common hepatic artery, and hepatogastric hiatus. The posterior aspect of the pancreatic head, liver, pelvis, small intestine, colon, and mesentery exhibited no abnormalities upon examination. The gallbladder was firstly removed intact. Subsequently, enlarged lymph nodes, fat and lymphatic tissue in the gallbladder triangle, hepatoduodenal ligament, and adjacent to the common hepatic artery were removed, and the hepatoduodenal ligament was skeletonized and cleared. Lymph nodes in the hepatogastric space were cleared, followed by complete hemostasis. To ensure a wide (>1 cm) resection margin, Intraoperative ultrasound was employed to determine the spatial relationship between the tumor and the hilar and intrahepatic vessels, leading us to conclude that anatomical hepatectomy was viable. Therefore, segment IV, V and VIII hepatectomy was performed. Hepatic parenchyma resection was performed with ultrasonic knife. After hepatic tumor resection, suture ligation or electrocautery was used to control residual bleeding sites. The surgical procedure proceeded smoothly, lasting 300 minutes, with an estimated blood loss of 100 ml. The surgical resection specimen is displayed in **Figures 4A, B**. The tumor was seen to consist of two parts, measuring approximately 6.5 x 2.5 x 2.5 cm and 2.6 x 2 x 0.8 cm, and was greyish-white in color.

## Prognosis and follow-up

The postoperative pathological examination using hematoxylin and eosin (HE) staining (**Figure 4C**) revealed extensive sampling (23 pieces) of the original tumor bed area, all of which showed tumorous extensive necrosis, peri necrotic fibrosis with inflammatory cell infiltration, consistent with post-treatment changes, and no active tumor components were seen. The tumor measured 6.6x2.5x2.5 cm with peritumoral invasion and no significant cancer thrombus was seen in the vessels. The surgical margins were clear. There were no metastases in the lymph nodes of groups 8 and 12A, 12B, 12P (0/3) and no metastases in the left gastric lymph node (0/7). The gallbladder showed chronic cholecystitis (**Figure 4D**).

This patient was discharged 19 days postoperatively (14 April 2023) after an uneventful postoperative course. After surgery, GEMOX chemotherapy (gemcitabine 1000 mg/m2, iv, d1, d8/3w in combination with oxaliplatin 125 mg/m2, iv, d2/3w) and anti-PD-1 immunotherapy (Tislelizumab 200 mg, iv, q3w) in combination with targeted therapy (Lenvatinib 8 mg, orally, qd) were continued on 4 July 2023 as a treatment regimen and continued until disease progression. Six cycles of combined chemotherapy were performed after operation. During the first-year post-surgery, follow-up visits were conducted monthly, encompassing physical examinations, routine blood tests, blood biochemistry analyses, tumor marker assessments, and abdominal MRI was reviewed every three months. The patient’s timeline, including initial diagnosis, combination therapy, radical surgery, and follow-up, is depicted in **Figure 3D**. As of March 2024, the patient shows no signs of local recurrence or distant metastasis of the tumor (**Figure 3E**).

## Discussion

Cholangiocarcinoma presents as an aggressive tumor with a bleak prognosis, posing significant challenges in treatment strategies. Studies have shown that the age-standardized mortality rate for overall patients with ICC has increased by 36.5% in men and 36.2% in women in 13 European Union countries, with similar increases in the United States and Australia (15). The NCCN guidelines categorize intrahepatic cholangiocarcinoma (ICC) into resectable, unresectable, and metastatic disease subtypes, with therapeutic considerations tailored accordingly (16). Surgery stands as the cornerstone treatment for intrahepatic cholangiocarcinoma (ICC), aiming to attain R0 resection while ensuring preservation of sufficient function in the future liver remnant (FLR). Nonetheless, only a minority of individuals with early-stage disease, approximately 35%, meet the criteria for surgical resection therapy (17). In our case, the tumor invaded the intrahepatic blood vessels and could not undergo radical surgery. For patients with advanced inoperable ICC, several therapeutic options are available, including systemic and targeted therapy, locoregional therapy, and radiotherapy. The NCCN guidelines advocate for cisplatin/gemcitabine (Gem/CDDP) combination chemotherapy as the preferred first-line treatment option. The ABC-02 trial, published in 2010, demonstrated that patients receiving Gem/CDDP chemotherapy for advanced biliary tract cancer achieved a median overall survival (OS) of 11.7 months (18). Oxaliplatin in combination with gemcitabine (GEMOX) is another common treatment regimen. The study by Fiteni F et al. included 771 and 699 patients with advanced ICC treated with Gem/CDDP and GEMOX, and showed that the weighted median survival for the treatment in the Gem/CDDP group and the GEMOX group was 9.7 months and 9.5 months, respectively. Survival outcomes were comparable between the two groups; however, Gem/CDDP chemotherapy was notably linked to a higher incidence of grade 3 and 4 adverse events, including malaise, diarrhea, hepatotoxicity, and hematotoxicity (19). In numerous cancer institutions, GEMOX chemotherapy is frequently favored as the first-line chemotherapy regimen. Zhu et al. included 53 ICC patients who received PD-1 inhibitor combined with lenvatinib and Gemox chemotherapy for a retrospective study, and all patients experienced grade 3 or 4 adverse events, including fatigue and bone marrow suppression (20). Shi et al.’s Phase II clinical study included 30 advanced stage ICC patients receiving gemcitabine and oxaliplatin combined with toripalimab and lenvatinib treatment. As a result, 56.7% of patients experienced grade 3 or higher adverse events, mainly bone marrow suppression (21). In our case, the patient also experienced adverse events of ≥ 3 levels, such as neutropenia and leukopenia, consistent with previous studies. In addition, our patient experienced liver toxicity, with alanine aminotransferase reaching a maximum of 246U/L (normal range of 7–40 U/L). Through appropriate drug intervention, these adverse events were effectively controlled and improved without affecting the treatment plan.

Median overall survival with chemotherapy alone still did not exceed 1 year, an unsatisfactory result. Cancer cells often exploit PD-1 signaling to evade immune surveillance (22). ICIs exert their anti-tumor effects by targeting cytotoxic T-lymphocyte-associated protein 4 (CTLA-4), PD-1 and PD-L1 (23). Chemotherapy may improve immunotherapeutic efficacy by destroying tumor tissue, overcoming immune rejection, and facilitating cross-presentation of tumor antigens (24–26). On 21 December 2022, Europe (EU) approved Durvalumab plus Gem/CDDP chemotherapy as a first-line treatment regimen for patients with advanced BTC. A phase II clinical trial conducted by Chen, Xinni et al. showcased that Camrelizumab, when combined with FOLFOX4 or GEMOX as first-line treatment, exhibited efficacy and tolerability in Chinese patients with advanced BTC. The study reported median progression-free survival of 5.3 months (95% CI = 3.7–5.7) and median overall survival of 12.4 months (95% CI = 8.9–16.1) (27). In a study by Ueno et al., it was suggested that patients with unresectable or recurrent biliary tract cancer (BTC) who underwent their initial chemotherapy treatment with Nivolumab in combination with Gem/CDDP chemotherapy achieved a median progression-free survival of 4.2 months (90% CI 2.8–5.6) and a median overall survival of 15.4 months (90% CI 11.8 - not estimable) (28). The aforementioned studies demonstrate favorable anti-tumor efficacy and manageable safety profiles in patients with advanced unresectable or metastatic BTC.

In recent years, significant advancements have been achieved in comprehending the molecular biology of ICC and in the development of pertinent targeted therapies. Lenvatinib, a multi-targeted tyrosine kinase inhibitor, finds utility as monotherapy or in combination with other anticancer agents in the treatment of radioiodine-refractory differentiated thyroid cancer, hepatocellular carcinoma, renal cell carcinoma, and endometrial cancer (29). Combining ICIs with TKIs is firmly supported by a robust biological rationale (30, 31). Lenvatinib may eliminate ICC cells through immunogenic cell death, reduce the number of cells targeted and destroyed by immune cells, and improve the efficacy of immunotherapy (32). Faiz et al.’s study showed that PD-1/PD-L1 is overexpressed in ICC (33), while Tian et al. further confirmed that the high expression of PD-1 in ICC tissue is associated with better OS (34), possibly due to PD-L1 inhibiting macrophage activity and survival rate, while PD-L1 antibodies can enhance the ability of macrophages to secrete inflammatory cytokines while promoting T cell proliferation and activation (35). Zhou et al. conducted a phase II clinical trial comprising 30 patients diagnosed with pathologically confirmed advanced intrahepatic cholangiocarcinoma (ICC). These patients were administered Lenvatinib in conjunction with GEMOX chemotherapy and the anti-PD-1 antibody Toripalimab as first-line therapy. The study reported a median progression-free survival (PFS) of 10.0 months, and the median overall survival (OS) was not reached. The objective response rate (ORR) was recorded at 80% (36). In a phase II clinical trial conducted by Zhu et al., 57 patients diagnosed with advanced biliary tract cancer (BTC) were treated with Lenvatinib in combination with a PD-1/PD-L1 inhibitor and GEMOX chemotherapy. The study reported a median overall survival of 13.4 months (95% CI: 10.0-NA) and a median progression-free survival of 9.27 months (95% CI: 7.1–11.6) (37). **Table 1** shows the research results of ICIs combined with immunotherapy and chemotherapy drugs for the treatment of advanced BTC. These studies suggest that the integration of immunotherapy with targeted therapy and systemic chemotherapy holds promise in providing therapeutic benefits for patients with advanced BTC achieve relatively good ORR, and further large-scale clinical trials are still warranted to validate the efficacy of the combination regimen. In the study presented in **Table 1**, the number of patients who achieved R0 surgical resection following conversion therapy was limited. Surgical resection is not the ultimate goal of conversion therapy; rather, the ultimate goal is for patients to achieve long-term survival and a high quality of life. Therefore, in terms of selecting the timing of surgery, we believe that if the treatment regimen is effective, conversion therapy should be allowed to reach a certain depth before surgery. Efforts should be made to achieve imaging complete response (CR) or more than 90% tumor necrosis before surgery. For tumors that do not shrink after two reassessments or if AFP levels do not decrease, timely surgical intervention should be performed. This approach aims to prevent postoperative tumor recurrence or metastasis due to insufficient depth of conversion therapy. During the treatment period, we rechecked blood tumor markers every 21 days and conducted liver MRI examinations every 2 months to evaluate changes in tumor size and morphology, thereby assessing the efficacy of the conversion therapy. In our report, the combination therapy had significant anti-tumor activity. After 8 cycles of treatment, the initially unresectable ICC was significantly smaller than before, and pathologically the tumor cells were completely necrotic and the structure disappeared. Patients were offered R0 resection with normalized tumor marker levels and no recurrence or distant metastases. If the tumor continues to grow during treatment, MDT discussion should be organized in time to change the treatment plan for patients to obtain the maximum benefit. Despite experiencing depression at the initial diagnosis of ICC, the patient ultimately found satisfaction with the treatment and demonstrated good compliance through our comprehensive therapeutic approach.

**Table 1:** Published trials and case reports of ICI combined with Targeted therapies.

Author	Country	Phase	Treatment Arm(s)	Patients(n=)	ORR (%)	PFS (Months)	OS (Months)	References
GuoMing Shi	China	II	Toripalimab+ Lenvatinib+GEMOX	30	80	10.2	22.5	(21)
Zhou Jian	China	II	Toripalimab+ Lenvatinib+GEMOX	30	80	10	–	(36)
Chengpei Zhu	China	II	ICIs+ Lenvatinib+GEMOX	57	43.9	9.27	13.4	(37)
H Li	China	II	Tislelizumab lenvatinib+GEMOX	25	56	–	–	(38)
Wei Zhang	China	–	Tislelizumab lenvatinib+GP	1	–	–	–	(14)
Zhuochao Zhang	China	–	Toripalimab +Pemigatinib+ GEMOX	1	–	–	–	(39)

This study has limitations. A single case is not enough to prove the universality and effectiveness of this treatment scheme in all patients with advanced ICC. To validate the efficacy of this combination therapy, conducting larger-scale clinical trials is imperative.

## Conclusion

In conclusion, the findings of the study indicate that immune and targeted combination chemotherapy exhibits favorable anti-tumor efficacy and safety in certain patients with advanced intrahepatic cholangiocarcinoma (ICC). This approach holds promise as a potential, feasible, and safe translational treatment strategy for advanced ICC. However, further large-scale clinical trials are necessary to validate these findings definitively.

## Data availability statement

The original contributions presented in the study are included in the article/supplementary material. Further inquiries can be directed to the corresponding author.

## Ethics statement

The studies involving humans were approved by the ethics committee of Wenzhou Central Hospital. The studies were conducted in accordance with the local legislation and institutional requirements. The participants provided their written informed consent to participate in this study. Written informed consent was obtained from the individual(s) for the publication of any potentially identifiable images or data included in this article.

## Author contributions

HZ: Writing – original draft, Writing – review & editing. H-bY: Writing – original draft, Writing – review & editing.
